# Identifying Molecular Targets of Lifestyle Modifications in Colon Cancer Prevention

**DOI:** 10.3389/fonc.2013.00119

**Published:** 2013-05-14

**Authors:** Molly M. Derry, Komal Raina, Chapla Agarwal, Rajesh Agarwal

**Affiliations:** ^1^Department of Pharmaceutical Sciences, Skaggs School of Pharmacy and Pharmaceutical Sciences, University of Colorado Anschutz Medical CampusAurora, CO, USA; ^2^University of Colorado Cancer Center, University of Colorado Anschutz Medical CampusAurora, CO, USA

**Keywords:** colorectal cancer, lifestyle modification, prevention, molecular targets, phytochemicals, silibinin, grape seed extract

## Abstract

One in four deaths in the United States is cancer-related, and colorectal cancer (CRC) is the second leading cause of cancer-associated deaths. Screening strategies are utilized but have not reduced disease incidence or mortality. In this regard, there is an interest in cancer preventive strategies focusing on lifestyle intervention, where specific etiologic factors involved in cancer initiation, promotion, and progression could be targeted. For example, exposure to dietary carcinogens, such as nitrosamines and polycyclic aromatic hydrocarbons influences colon carcinogenesis. Furthermore, dietary deficiencies could alter sensitivity to genetic damage and influence carcinogen metabolism contributing to CRC. High alcohol consumption increases the risk of mutations including the fact that acetaldehyde, an ethanol metabolite, is classified as a group 1 carcinogen. Tobacco smoke exposure is also a risk factor for cancer development; approximately 20% of CRCs are associated with smoking. Additionally, obese patients have a higher risk of cancer development, which is further supported by the fact that physical activity decreases CRC risk by 55%. Similarly, chronic inflammatory conditions also increase the risk of CRC development. Moreover, the circadian clock alters digestion and regulates other biochemical, physiological, and behavioral processes that could influence CRC. Taken together, colon carcinogenesis involves a number of etiological factors, and therefore, to create effective preventive strategies, molecular targets need to be identified and beleaguered prior to disease progression. With this in mind, the following is a comprehensive review identifying downstream target proteins of the above lifestyle risk factors, which are modulated during colon carcinogenesis and could be targeted for CRC prevention by novel agents including phytochemicals.

## Introduction

It is projected that by 2030 the number of new cancer cases will increase by 70% worldwide due to demographic changes alone; the significant rise is attributed to adoption of western lifestyle habits (Franceschi and Wild, 2013). Globally colorectal cancer (CRC) is the second most common cancer in women and the third most common in men; according to the American cancer society (ACS) in the United States alone, there are estimated to be approximately 50,830 deaths associated with CRC in 2013. CRC diagnostic screening strategies are available, however, compliance is low, and conventional treatments result in severe toxicity and do not decrease disease incidence (Diaz et al., 2006; Bretthauer, 2010). With this in mind, prevention strategies need to be developed and implemented within the community, based on existing knowledge about risk factors and natural CRC disease progression. There is extensive research examining the risk factors for CRC; including high red meat consumption, high fat low fiber diet, alcohol and tobacco consumption, obesity, lack of physical activity, and sleep deprivation (Huxley et al., 2009; Wei et al., 2009; Basterfield and Mathers, 2010; Chan and Giovannucci, 2010; Thompson et al., 2011). These lifestyle factors are the major cause of disease burden globally, and combined with physician’s tendency to prescribe medication rather than prescribing a healthy lifestyle, have resulted in a global health crisis (Yousefi et al., 2012; Franceschi and Wild, 2013). However, advancing knowledge investigating the molecular basis of carcinogenesis has created a remarkable opportunity to develop effective prevention strategies through lifestyle modification. Identifying molecular targets of lifestyle modification allows researchers to develop effective non-toxic interventions, further allowing physicians to design personalized lifestyle prescriptions based on patient history, resulting in improved patient health and well-being.

## Identifying Molecular Pathways Involved in CRC Etiology

Colorectal cancer is a complex multi-factorial disease; prior to diagnosis, there are decades of complex genetic and environmental interactions that ultimately lead to disease initiation, promotion, and progression (Nambiar et al., 2010). The cellular environment plays a large role in the disease, and as early as 1863, it was hypothesized that the origin of cancer was at sites of chronic inflammation (Balkwill and Mantovani, 2001). During the normal inflammatory response, anti-inflammatory cytokine production follows the production of pro-inflammatory cytokines, creating a balanced environment. Chronic inflammation can be the result of persistent initiating factors in the surrounding environment, or faulty repair mechanism resulting in unrepaired damage, ultimately leading to neoplasia (Coussens and Werb, 2002).

During chronic inflammation, imbalances between the production of reactive oxygen species (ROS) and the detoxification of these reactive species results in oxidative stress with in the target tissue; this stress can result in DNA damage and further reduce DNA repair (Coussens and Werb, 2002). On the other hand, cells can inherit changes in phenotype, independent of alterations in the DNA; termed epigenetics, more specifically chromatin based events that regulate the DNA template (Dawson and Kouzarides, 2012). Further alterations in gene methylation and acetylation status result in altered chromatin regulators and gene expression (Dawson and Kouzarides, 2012). These epigenetic changes have been associated with oncogene modification or abnormal expression patterns that can lead to the induction and maintenance of various cancers (Dawson and Kouzarides, 2012). At the same time, the tumor microenvironment can also alter the DNA template resulting in modified protein translation, function, and transport (Xu et al., 2005).

Once the cells are transformed, the extent to which inflammatory cell populations infiltrate the tissue depends on the balance of cytokines released from the tumor microenvironment. Inflammatory cells are able to produce a wide array of cellular signals including: ROS, proteases [i.e., MMPs (matrix metalloproteinase)], soluble cell death ligands [i.e., TNF-α (tumor necrosis factor alpha)], interleukins (ILs) (i.e., IL-6), and interferons (IFNs) (Coussens and Werb, 2002). NFκB is a central mediator of the immune response that further regulates inducible-nitric oxide synthase (iNOS) which is an enzyme that catalyzes the formation of nitric oxide (NO), leading to inflammatory and hormone signaling modulation (Fujimoto et al., 2005). Additionally, signaling lipid mediators, specifically prostaglandins (PGs), are formed during oxidation of arachidonic acid (AA) via cyclooxygenases (COX-1 and COX-2) which are involved in numerous processes including: inflammation, hormone regulation, and cell growth (Vane et al., 1998). Inflammatory mediators are also involved in the regulation of various growth factors and hormones, including VEGF (vascular endothelial growth factor) and IGF-1 (Insulin-like growth factor-1), which are involved in tumor cell proliferation and metastasis (Akagi et al., 1998).

The cells’ response to the above stimuli depends on its ability to adapt and continue on the path of cell survival, allowing cells to repair DNA damage and continue through the cell cycle, or direct toward the path of programed cell death (PCD). Hallmarks of colon carcinogenesis are uncontrolled cellular proliferation and resistance to cell death; therefore, strategies that focus on these processes would be of clinical significance. Proliferative signaling pathways often begin with activation of a receptor tyrosine kinase (RTK) (i.e., EGFR, epidermal growth factor receptor) by a growth factor and subsequent downstream protein activation (i.e., MAPK, mitogen-activated protein kinase) that can lead to activation of multiple pathways involved not only in proliferation but also cell cycle regulation and cellular metabolism (Fritz and Fajas, 2010). Proliferation is controlled by the cell cycle and involves DNA replication and cellular division; this process is controlled by a cascade of protein phosphorylation events and a set of protein checkpoints that can arrest the cell at specific stages of the cycle (Collins et al., 1997). PCD is an intracellular process that refers to apoptosis, autophagy, and programed necrosis. The process of apoptosis is highly complex involving two main pathways, the extrinsic or death receptor pathway (i.e., DR4/DR5) and the intrinsic or mitochondrial-derived pathway (Elmore, 2007). Autophagy is a lysosomal-dependent pathway involving degradation and recycling of cellular components; controlled via an autophagy-related gene network (Liu et al., 2012b). Programed necrosis or necroptosis is a result of the initiation of different receptor-sensor complexes via various stimuli, including TNF-α and ROS species (Han et al., 2011). Colorectal carcinogenesis involves initiating events resulting in genomic alteration, which leads to neoplastic growth, resulting in the alteration of microenvironment, and further tumor promotion from adenoma to adenocarcinoma, ultimately resulting in disease progression or metastasis, via internal and external cellular stimuli. A wide variety of cellular pathways are involved through out the colorectal carcinogenesis process, thus, identifying molecular targets that are altered in response to lifestyle choices is key in understanding how to effectively prevent CRC.

## Biological Responses of Meat Consumption in Relation to CRC Risk

Humans over a long period of time have adapted to consuming large amounts of lean red meat; for many adults in the US, red meat is part of a habitual diet with average consumption of red meat per day being 128 g (Mann, 2000; Daniel et al., 2011). However, numerous epidemiological studies suggest an association between red and processed meat consumption and the risk of CRC (Chan et al., 2011). Red meat is considered the intake of mutton, lamb, veal, pork, and beef, and processed meat is defined as the total intake of cured or preserved meats, sausages, ham, and bacon (Chan et al., 2011). To develop prevention strategies that will reduce CRC risk, we need to further understand the role of these factors in colorectal carcinogenesis.

**Figure 1:**
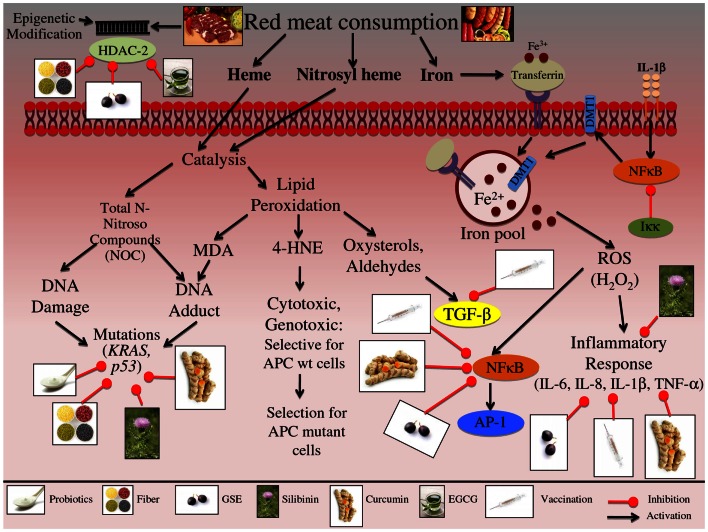
**Effect of red meat consumption on signaling pathways involved in colon carcinogenesis**. Consumption of red meat has been shown to induce epigenetic changes in host DNA. These changes occur specifically through altering the levels of histone deacetylase-2 (HDAC-2). Red and processed meat further contains iron, heme, and nitrosyl heme, all of which at high levels may increase the risk of CRC development. Both heme and nitrosyl heme undergo catalysis resulting in the formation of *N*-Nitroso compounds (NOC); these NOCs can either result in DNA damage or DNA-adduct formation. Red meat consumption specifically leads to mutations in *p53* and *KRAS* genes, further leading to the initiation and progression of colon carcinogenesis. Alternatively, heme catalysis can also lead to generation of lipid peroxidation end products, such as malondialdehyde (MDA), 4-hydroxynonenal (4-HNE), oxysterols, and aldehydes. MDA exposure can result in DNA-adduct formation, leading to DNA mutations and aberrant proliferation, further contributing to the initiation of CRC. 4-HNE is cytotoxic and genotoxic compound, that targets colon cells that carry a wild-type APC gene; this selective toxicity results in enhancement of colon cells that carry a mutated APC gene, resulting in CRC promotion and progression. Additionally lipid peroxidation results in the formation of oxysterols and aldehydes, which further alter hormone signaling, specifically TGF-β, ultimately resulting in uncontrolled proliferation that contributes to the promotion and progression of CRC. Another major component of red meat is iron, ferric iron (Fe^3+^) binds to transferrin, resulting in receptor activation and endocytosis. Ferric iron is further converted to ferrous iron (Fe^2+^) via divalent metal transporter 1 (DMT1), which then contributes to the cell’s overall iron pool. Iron has been linked to the production of reactive oxygen species (ROS), specifically H_2_O_2_; these reactive species can then up regulate inflammatory mediators, such as NFκB, IL-6, IL-8, IL-1β, and TNF-α, leading to the promotion and progression of CRC. Furthermore IL-1β signaling up regulates NFκB, which then activates DMT1 iron transporters, resulting in increased levels of ferrous iron within the cell, representing a feed-back loop in iron regulation. The illustration also denotes the modulatory role of lifestyle interventions at various levels on these aberrant signaling pathways. The photographs of lifestyle interventions were sourced and adapted from http://www.freedigitalphotos.net/.

Consumption of red and processed meats may induce several biological responses that could be responsible for the increased risk of CRC development as shown in Table 1 and Figure 1. White meat is not associated with an increased risk of CRC, so what dietary factor/s in red meat increases the risk of cancer development? One important dietary factor could be heme, an iron porphyrin pigment of red meat, present 10-fold higher in red meat compared to white meat (Schwartz and Ellefson, 1985; Ishikawa et al., 2010). A free ferrous iron is released when heme oxygenase-1 (HO-1) resolves dietary heme in the intestinal mucosa (Ishikawa et al., 2010). Iron itself has been linked to the increased production of ROS, specifically H_2_O_2_, which can further induce genetic mutations, inflammatory mediators, and other cytotoxic effects (Klaunig and Kamendulis, 2004; Knobel et al., 2007). H_2_O_2_ more specifically induces IL-8, IL-6, IL-1β, and TNF-α cytokine production, and can further activate NFκB and AP-1 transcription factors promoting a pro-inflammatory signal (Morcillo et al., 1999; Haddad et al., 2001; Yamamoto et al., 2003).

**Table 1:** **Molecular targets of lifestyle modification**.

	Detox pathways	Oxidative stress	Inflammation	DNA repair	Epigenetic mechanisms	Oncogene alteration	Hormone signaling	Proliferation	Apoptosis	Cell cycle	Metastasis
Red meat		H_2_O_2_	IL-8, IL-6, IL-1β, TNF-α, NFκB, AP-1	p53	HDAC-2	APC, p53	TGF-β	TGF-β, AP-1	p53	AP-1, p-53	Notch
Alcohol	CYPs, catalase	NO, HIF-1α, NADPH oxidase	TNF-α, IL-6	p53	SAM, *p16*	p53	EGFR	AP-1, PI3K/AKT, EGFR, ERK 1/2	P53, PI3K/AKT	AP-1, p16, p-53	VEGF, MMP-2, MMP-7, MMP-9
Tobacco	CYPs, GSTs, UGTs	ROS	NFκB, COX-2	OGG1, APEX1/APE1, XRCCI	*KRAS/BRAF*	C-myc	EGFR	MAPK, AP-1, EGFR	BCL-2, Bad, MAPK	AP-1	MMP-2, VEGF
Physical activity/obesity	SOD, Nrf2, GSTs	ROS, Nrf2	TNF-α, IL-6, iNOS, NFκB, COX-2			C-myc, β-catenin	YY, GLP-1, PP, leptin, insulin IGFBP-3, PPARα, GSK3β	Leptin, MAPK, PI3K/AKT, PCNA, β-catenin, GSK3β	Leptin, MAPK, PI3K/AKT	Cyclin D	VEGF
Circadian clock	CYPs, HLF, E4BP4	TfR1, ROS	NFκB	p53		C-myc, β-catenin		P53, β-catenin	p53, p21 BCL-xL, Bim, cleaved caspase-3 and -9	*p21, Wee1*, Cyclin D1/E, p53, p21	VEGF

Another dietary factor that could contribute to CRC risk is malondialdehyde (MDA), a known mutagen, which is formed during lipid peroxidation and found at high levels in the plasma when beef is consumed compared to chicken (Basu and Marnett, 1983; Toden et al., 2010). MDA exposure results in colonic DNA damage, and studies have shown that there is a dose-dependent positive association between MDA plasma levels and the extent of DNA signal-strand breaks (SSB) and double-strand breaks (DSB) (Toden et al., 2010). Additionally, red meat consumption leads to the production of *N*-nitroso compounds (NOC) in the large bowel leading to the DNA-adduct formation through the binding of telomere stabilizing proteins (i.e., TRF2) (O’Callaghan et al., 2012). Moreover, red meat consumption initiates epigenetic changes in host DNA; genotoxic exposure results in modification of NuRD histone deacetylase complexes (HDAC) that control transcriptional repression, including HDAC-2 (Feng and Zhang, 2003; Hebels et al., 2012). Furthermore, animal-fat oxidation occurs when red meat is consumed, resulting in formation of oxysterols and aldehydes that can alter hormone signals such as TGF-β; these external stimuli can result in uncontrolled proliferation (Biasi et al., 2008). Apart from these changes, genes that are involved in cancer metastasis are also modified in response to red meat exposure; specifically, the Notch signaling pathway, which is abnormally activated in CRC and plays a key role in epithelial-mesenchymal transition (EMT) (Bolos et al., 2007; Wang et al., 2010; Hebels et al., 2012).

## Identifying Molecular Targets of Alcohol-Induced CRC

Alcohol (ethanol) by virtue of its oral consumption, not only reaches the gastrointestinal tract but also reaches every cell of the body, and is a major risk factor for the development as well as progression of various forms of cancer including CRC (Boffetta and Hashibe, 2006; Haas et al., 2012). Alcohol is acknowledged as a carcinogen by the World Health Organization (WHO) and the International agency for Research on Cancer (IARC), and its intake is categorized into less than 12 g of alcohol, moderate alcohol intake (12–35 g/day), and high alcohol intake (>35 g/day). One unit of alcohol is one 12 oz beer, 4 oz of wine, or 1.5 oz of liquor, which is equivalent to approximately 7.9 g of alcohol (Kontou et al., 2012). During metabolism, alcohol dehydrogenase (ADH), catalase, and cytochrome P450 subenzyme 2E1 (CYP2E1) catalyze the oxidation of alcohol to acetaldehyde (AA), a group I carcinogen (Haas et al., 2012; Mikko, 2012). The mechanisms involved in alcohol-induced cancer progression are difficult to resolve due to the limitations involved in determining individual risks, because in most cases smoking and chronic inflammation are additional risk factors common in patients that over consume alcohol (Haas et al., 2012). Developing effective prevention strategies requires knowledge of the existing etiologic factors that contribute to alcohol-induced carcinogenesis.

**Figure 2:**
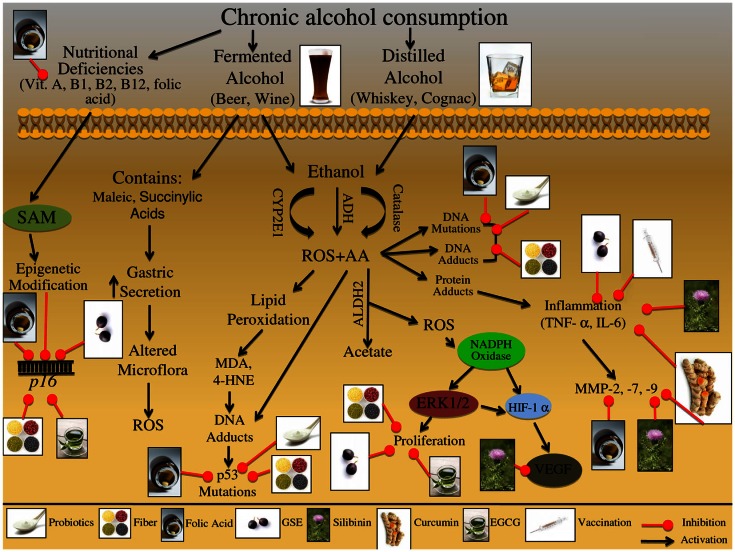
**Effect of chronic alcohol consumption on the growth and development of colon carcinogenesis**. Chronic consumption of alcohol leads to deficiency of vitamins-A, B1, B2, B12, and folic acid. These deficiencies can further lead to alterations in epigenetic regulators; specifically, folate deficiency leads to altered *S*-adenosylmethionine (SAM) synthesis, resulting in altered *p16* gene expression. These epigenetic modifications are involved in the initiation and promotion of CRC. Beer consumption can lead to increased reactive oxygen species (ROS). Specifically, maleic and succinylic acids that are found in beer can result in increase gastric secretion; this increase in acidic environment leads to alteration in the microflora present in the gut, ultimately resulting in increased ROS production. Consumed ethanol is metabolized via oxidation to acetaldehyde (AA); this metabolism is mediated via alcohol dehydrogenase (ADH), cytochrome P450 subenzyme 2E1 (CYP2E1), and catalase. Additional metabolic byproducts of ethanol metabolism include ROS, which are released during the CYP2E1 oxidation reaction; these reactive intermediates enhance lipid peroxidation leading to DNA-adduct formation. Adduct formation can then result in DNA mutations, specifically in the *p53* gene, which contributes to the initiation and promotion of CRC. AA alone is a mutagenic compound known to form adducts with DNA and proteins, as well as, induce DNA mutations; furthermore protein adduct formation has been shown to induce inflammatory responses via TNF-α and IL-6. These inflammatory mediators further regulate the expression of matrix metalloproteinase MMP-2, -7, and -9, which are involved in the promotion and progression of CRC. Additionally AA can be oxidized by aldehydes dehydrogenase 2 (ALDH2) resulting in acetate and ROS formation; these ROS species have been indicated in the activation of NADPH oxidase. NADPH oxidase is an enzyme complex that further modulates downstream effectors proteins, such as ERK1/2 and HIF-1α signaling; this down stream protein modification results in alteration of proliferative and metastatic signaling pathways, which are involved in CRC promotion and progression. The illustration also denotes the modulatory role of lifestyle interventions at various levels on these aberrant signaling pathways. The photographs of lifestyle interventions were sourced and adapted from http://www.freedigitalphotos.net/.

The numerous molecular pathways that are involved in alcohol exposure and CRC etiology that reviewed in Table 1 and Figure 2. Chronic alcohol consumption results in nutritional deficiencies, specifically decreases absorption of folic acid and vitamins B1, B2, and B12; allowing the cells to become vulnerable to the increase in ROS resulting in oxidative stress, this environmental change results in variety of pathway alterations within the cell (Testino, 2011). Furthermore, fermented alcoholic beverages, such as beer and wine, contain compounds that contribute to ROS generation; specifically maleic and succinylic acids that stimulate gastrin-mediated secretion potentially altering the colon microflora resulting in increased NO production (Haas et al., 2012). Additionally, Vitamin A synthesis is mediated via the CYP2E1 enzyme, which also metabolizes alcohol; this substrate competition induced from chronic alcohol consumption results in decreased Vitamin A levels (Testino, 2011). Decreased vitamin A levels result in decreased expression of the *AP-1* gene, which is involved in cell cycle regulation and inflammation (Testino, 2011). Likewise, alcohol consumption results in increased inflammation, as demonstrated by increased secretion of inflammatory mediators, such TNF-α and IL-6, when CRC cells are exposed to alcohol (Zhao et al., 2004; Amin et al., 2009). Moreover, as a result of long-term alcohol consumption, folate levels are decreased, further altering the synthesis of *S*-adenosylmethionine (SAM), a key methyl donor involved in epigenetic alterations leading to modification of gene expression (Sauer et al., 2010). Similarly, alcohol exposure has been shown to alter the histone methylation pattern of the *p16* gene promoter, resulting in decreased p16 protein levels and uncontrolled cell cycle regulation (Sauer et al., 2010). In addition, accumulation of intracellular ROS leads to the induction of NADPH oxidase and downstream pathways such as hypoxia-inducible factor-1α (HIF-1α) signaling; leading to up regulation of PI3K/AKT and VEGF signaling, which are involved in apoptotic and metastatic signaling (Morgensztern and McLeod, 2005; Wang et al., 2012a). Likewise more proliferative and metastatic pathways are modulated by alcohol consumption; these include MMP-2, MMP-7, MMP-9, EGFR, and ERK 1/2, which in turn promote proliferative and EMT transition pathways (Forsyth et al., 2010).

## Molecular Mechanisms of Tobacco-Induced Colorectal Carcinogenesis

Everyday humans are exposed to a variety of toxic and carcinogenic compounds due to life style habits including smoking tobacco. It has been estimated that tobacco has killed more than five million people in 2008 and will be responsible for the death of more than eight million by 2030 (Lodovici and Bigagli, 2009). A wide variety of malignancies are associated with tobacco consumption, with the strongest associations seen not only in the respiratory tract, but the gastrointestinal and urogenital systems; it is estimated that approximately 20% of CRC cases can be attributed to tobacco exposure (Giovannucci and Martinez, 1996; Tsoi et al., 2009). The major classes of carcinogenic compounds in tobacco smoke are polycyclic aromatic hydrocarbons (PAHs), aromatic amines, nitrosamines, and heterocyclic amines (HCAs); these carcinogenic compounds can enter the alimentary tract or the circulatory systems (Fischer et al., 1990; Kasahara et al., 2008). With in the body these compounds are then metabolized by CYPs (CYP1A1, CYP1A2, CYP2E1, CYP2A6), leading to DNA-adduct formation or by glutathione *S*-transferases (GSTs) (GSTM1, GSTT1, GSTP1) leading to excretion (Barrowman et al., 1989; Guengerich and Shimada, 1991; Alexandrov et al., 1996; Koh et al., 2011). Identifying the molecular mechanisms involved in tobacco detoxification allows the development of effective lifestyle modification strategies to prevent CRC development in tobacco smokers.

**Figure 3:**
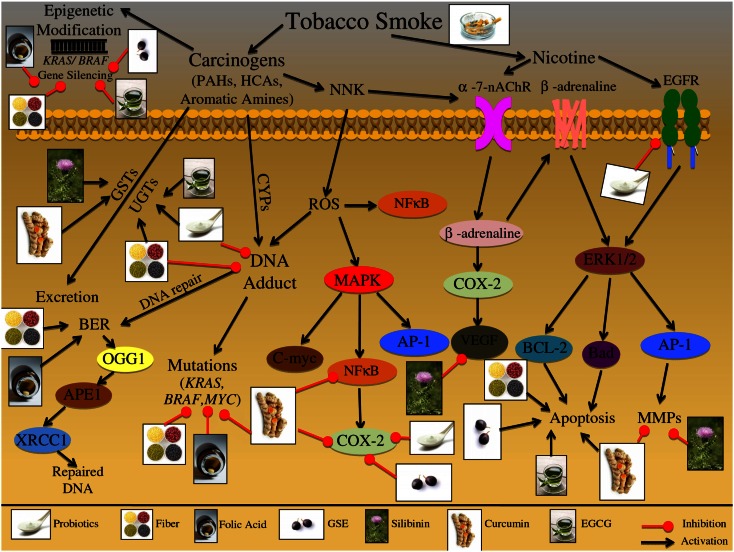
**Effect of cigarette smoke on the etiology of colon carcinogenesis**. Cigarette smoke contains nicotine as well as numerous carcinogenic compounds that effect the initiation, promotion, and progression of colorectal cancer (CRC). These carcinogenic compounds include polycyclic aromatic hydrocarbons (PAHs), heterocyclic amines (HCAs), and aromatic amines. These compounds have the ability to induce epigenetic changes in *KRAS* and *BRAF* genes; both these genes are important in preventing the initiation of CRC. Furthermore, these carcinogenic compounds can be bioactivated via cytochrome P450 subenzyme (CYPs); this activation can result in DNA-adduct formation, potentially resulting in DNA mutations in *KRAS*, *BRAF*, and *MYC* if the DNA damage is not repaired. DNA repair pathways such as the base excision repair (BER) pathway can reverse the damage induced by these carcinogens through up regulation of various repair proteins such as, OGG1, APEX1/APE1, and XRCC1. Alternatively these carcinogenic compounds can be excreted from the body via glutathione *S*-transferases (GSTs) and/or UDP-glucuronosyltransferases (UGTs). Specific carcinogenic compounds, such as nicotine-derived nitrosamine ketone (NNK) have been shown to induce the production of ROS; these reactive intermediates can then activate numerous molecular pathways including MAPK and NFκB. The MAPK signaling cascade has numerous potential protein targets, including NFκB, AP-1, and C-myc; the activation of these pathways can further result in increased inflammatory markers such as COX-2, leading to the initiation and promotion of CRC. Additionally, NNK can activate nicotinic acetylcholine receptors (nAChRs), resulting in β-adrenaline upregulation; β-adrenaline can up regulate COX-2 levels, further leading to VEGF upregulation. Alternatively β-adrenaline can bind and activate the β-adrenaline receptor leading to cascade activation; receptor stimulation triggers ERK1/2 activation leading to up regulation of downstream targets such as, BCL-2, Bad, and AP-1. The downstream targets of the β-adrenaline receptor lead to the induction of apoptosis and further activation of metastatic proteins such as MMPs. Additionally, cigarette smoke contains nicotine which can activate multiple cell membrane receptors including, nAChRs, β-adrenaline, and EGFR. Activation of these membrane receptors leads to the up regulation of inflammatory, apoptotic, and metastatic proteins, such as COX-2, Bad, BCL-2, AP-1, and VEGF. The illustration also denotes the modulatory role of lifestyle interventions at various levels on these aberrant signaling pathways. The photographs of lifestyle interventions were sourced and adapted from http://www.freedigitalphotos.net/.

There are multiple molecular pathways involved in tobacco exposure and CRC etiology that are reviewed in Table 1 and Figure 3. Nicotine exposure results in the activation of nicotinic acetylcholine receptors (nAChRs) and may contribute to cancer progression; coupled with the fact that as tumors progress there is an increased expression of nAChRs, further demonstrating the involvement of this pathway during carcinogenesis (Russo et al., 2012). Nitrosamines, such as nicotine-derived nitrosamine ketone (NNK), are high affinity ligands for the nAChR signaling and have been shown to increase intracellular ROS levels in human CRC cells (Ye et al., 2004). This oxidative stress triggers activation of inflammatory pathways including that of NFκB, which further acts as a positive regulator of COX-2 expression (Kosaka et al., 1994). There are additional biomarkers that indicate tobacco exposure; these biomarkers include oxidative DNA base modification or DNA-adduct formation, both of which have been shown to be elevated in colon tissue of smokers (Alexandrov et al., 1996; Kasahara et al., 2008; Lodovici and Bigagli, 2009). These events trigger DNA repair, specifically base excision repair (BER) pathway, which is one of the four major DNA repair pathways, involving multiple proteins such as OGG1, APEX1/APE1, and XRCC1 (Kasahara et al., 2008). Another potential mechanism involved in the initiation of tobacco-induced colorectal carcinogenesis could be epigenetic changes, resulting in *KRAS* and *BRAF* gene silencing that have been observed in the majority of the diagnosed CRC cases (Samowitz et al., 2006; Rosenberg et al., 2007).

Another class of receptors involved in nicotine signaling includes β-adrenoceptors, which can initiate a number of physiological responses, including metabolic and immunomodulatory responses (Civantos Calzada and Aleixandre de Artinano, 2001; Oberbeck, 2006). Once activated, these receptors increase inflammatory signals and metastatic mediators, such as COX-2 and MMP-2 (Schuller et al., 1999; Hori et al., 2011). Together with nAChR signaling, β-adrenoceptors have been linked to metastatic growth factors, such as VEGF (Lutgendorf et al., 2003; Wong et al., 2007). Chronic nAChRs and β-adrenoceptors activation due to tobacco smoke exposure has further been linked to apoptotic signaling; specifically via phosphorylation of pro-apoptotic proteins, BCL-2 and BAD (Heusch and Maneckjee, 1998; Jin et al., 2004). In addition, smoke inhalation initiates and promotes proliferative pathways, specifically MAPK signaling, which is activated in response to NNK exposure (Ye et al., 2004).

## Physiological Responses to Physical Activity and Obesity-Induced CRC

According to the Center for Disease Control (CDC) and the WHO, obesity and cancer are two major epidemics in this century, and prevalence has dramatically increased in the last few decades. High consumption of processed foods, animal fat, and a high calorie diet are risk factors for CRC development. Overweight is defined as those who have a body mass index (BMI) ≥25 kg/m^2^, obese individuals have a BMI ≥30 kg/m^2^; currently 66% of adults are overweight and 33% are obese (Flegal et al., 2012; Vucenik and Stains, 2012). In 2008, there were over 500 million people that were in the obese category, as a result of a chronic positive energy balance. This lack of equilibrium leads to the systemic secretion of various factors, such as TNF-α, IL-6, insulin, insulin-like growth factor-1 (IGF-1), adiponectin, and leptin, which play an important role in carcinogenesis including CRC (Harvey et al., 2012; Vucenik and Stains, 2012). Over the last 20 years, there has been a growing interest in the benefits of physical activity and cancer etiology; current physical activity guidelines recommend healthy adults to perform a minimum of 60-min moderate-intensity or 30-min of vigorous-intensity exercise daily to promote health, according to the American Institute of Cancer Research (AICR) (Wiseman, 2008). There are a number of observational studies examining prevention strategies with convincing evidence that physical activity reduces CRC risk, when comparing the most active with the least active individuals (Winzer et al., 2011). Repeated aerobic exercise has shown to decrease blood pressure, fasting glucose, insulin, and atherogenic lipids (Pedersen and Fischer, 2007). Currently, there are numerous pathways that are modified with physical activity that could target colon carcinogenesis; specifically, hormonal, inflammatory, angiogenic, apoptotic, and proliferative pathways (Winzer et al., 2011). Identifying biomarkers and further examining their mechanistic role in CRC initiation, promotion, and progression will, thus, further the development of physical activity interventions for the prevention of CRC.

**Figure 4:**
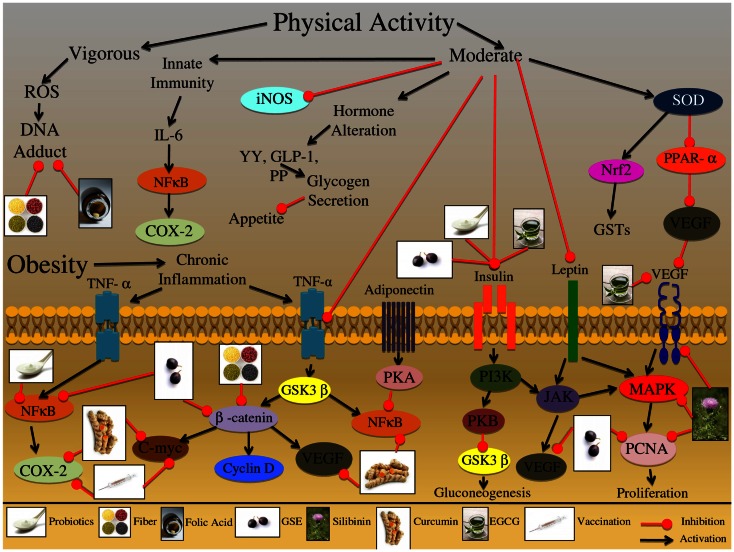
**Effect of physical activity and obesity on the growth and development of colon carcinogenesis**. Vigorous physical activity had been shown to induce the production of reactive oxygen species (ROS) leading to DNA-adduct formation, potentially affecting the initiation of colorectal cancer (CRC). Alternatively, moderate physical exercise has been shown to induce an innate immune response through IL-6 receptor activation and downstream protein up regulation. This adaptive immune response protects the organisms against chronic inflammatory conditions, which are known to increase the risk of CRC development. Further, moderate physical activity has been shown to inhibit the expression of other inflammatory mediators, such as iNOS, which may play a role in chronic inflammatory conditions. Moderate physical activity has been also shown to alter specific hormones that are related to appetite and satiety signals; these hormones include YY, GLP-1, and PP, all of which are involved in glycogen secretion. Practicing moderate physical activity also results in the up regulation of superoxide dismutase (SOD); this enzyme further activates detoxification pathways via up regulation of the Nrf2 proteins and GSTs enzymes. Additionally, SOD inhibits PPAR-α leading to the reduced expression of VEGF, which is involved in the promotion and progression of CRC. Obesity is a result of an energy imbalance that results in aberrant activation of various receptor proteins involved in inflammation, proliferation, and hormone regulation. This abnormal activation creates an environment that is chronically inflamed and can result in the initiation, promotion and progression of CRC. One of the major inflammatory mediators is TNF-α, which is activated at the cell membrane, resulting in downstream protein cascade activation. TNF-α activation can result in induction of NFκB and COX-2 signaling which initiates an inflammatory response. Additionally, the TNF-α receptor pathway can trigger activation of the GSK3β enzyme; this enzyme regulates numerous proteins, including β-catenin and NFκB. β-catenin further regulates other proteins that are involved CRC development; these proteins include C-myc, cyclin D, and VEGF, which are involved in oncogene signaling, cell cycle regulation, and metastatic development. Adiponectin is another factor that is highly expressed in obese individuals, which has been shown to increase glucose uptake and fatty acid oxidation; once the adiponectin receptor is activated it results in PKA kinase upregulation, which further inhibits NFκB in adipocytes. This inhibition ultimately leads to down regulation of cell adhesion molecules that are important regulators of CRC promotion and progression. Insulin signaling is another important pathway that is chronically up regulated in obese individuals; binding of insulin to its receptor triggers a conformational change that results in the activation of downstream kinase targets such as, PI3K. PI3K has the ability to trigger PKB kinase activation, which ultimately results in increased glycogen secretion and decreased gluconeogenesis; alternatively PI3K can activate the JAK protein kinase, which plays a role in immune function and inflammatory responses. Another factor that has been shown to activate the JAK protein is leptin; this factor is secreted from adipose tissue and in addition to activating JAK it has also been shown to activate the MAPK signaling cascade. Furthermore, both JAK and MAPK signaling pathways are involved in the regulation of proliferative and metastatic associated molecules, such as PCNA and VEGF; both of which are important regulators of CRC promotion and progression. The illustration also denotes the modulatory role of lifestyle interventions at various levels on these aberrant signaling pathways. The photographs of lifestyle interventions were sourced and adapted from http://www.freedigitalphotos.net/.

Additionally, Table 1 and Figure 4 is a review of the numerous cellular pathways that are affected by physical activity and obesity, which are further involved in CRC etiology. During exercise, a 10- to 40-fold increase in oxygen uptake occurs relative to the resting state which can cause an increased ROS formation (Na and Oliynyk, 2011). It has been reported that vigorous exhausting exercise increases DNA damage, while moderate exercise does not increase, but instead, alleviates the oxidative stress and DNA damage (Poulsen et al., 1999; Sato et al., 2003). Examining detoxification pathways during physical activity revealed that superoxide dismutase (SOD) was up regulated, as well as, downstream protein players, such as nuclear transcription factor erythroid 2p45 (NF-E2)-related factor (Nrf2) (Asghar et al., 2007). Nrf2 then translocates to the nucleus to initiate transcription of additional detoxifying enzymes, such as GSTs (Asghar et al., 2007). Physical activity has the ability to increase energy expenditure and decrease energy intake through hormone modulation; acute exercise results in increased levels of certain satiety hormones, specifically polypeptide YY, GLP-1, and PP, all of which are involved in glycogen secretion, the major energy source in the body (Martins et al., 2008). Leptin is another example of the ability of physical activity to modulate hormones-leptin levels decrease with moderate exercise; this hormone is synthesized by adipocytes and is important in regulating food intake (Ahima et al., 1996). Furthermore, leptin is involved in proliferative and apoptotic signaling, specifically activation of MAPK and PI3K/AKT pathways (Cirillo et al., 2008). Correspondingly, proliferative marker such as proliferating cell nuclear antigen (PCNA) is down regulated during moderate physical activity (Demarzo et al., 2008).

The increase of adipose tissue observed in obese individuals results in systemic low-grade inflammation, via up regulation of TNF-α; this sustained up regulation can lead to chronic inflammatory conditions that elevate the risk of CRC development (Drevon, 2005). However, with regular exercise, the anti-inflammatory effects of acute bouts of exercise protect against chronic systemic low-grade inflammation. Physical activity has been shown to increase inflammatory mediators such as IL-6 (Kim et al., 2009; Brandt and Pedersen, 2010). It is speculated that this increased IL-6 production during exercise may work in a endocrine-type fashion and increase hepatic glucose production or lipolysis in adipose tissue (Pedersen and Febbraio, 2008). It is hypothesized that induction of the adaptive immunity through moderate exercise can ultimately prevent chronic inflammatory conditions. Furthermore, physical activity was able to decrease the expression of iNOS and TNF-α in the plasma and colon mucosa, supporting an overall anti-inflammatory role of moderate exercise (Na and Oliynyk, 2011). Physical activity was also shown to significantly decrease the IGF-1/Insulin-like growth factor-binding protein 3 (IGFBP-3) ratios and aberrant β-catenin signaling (Ju et al., 2008). An inappropriate stabilization, translocation, and activation of β-catenin are hallmarks of sporadic and familial CRC. Thus, physical activity modulation of β-catenin results in downstream modulation of oncogenic target genes such as c-myc and VEGF (Zhang et al., 2001; Barker and Clevers, 2006; Saif and Chu, 2010). Moreover, peroxisome proliferator-activated receptor-α (PPARα) is a metabolic regulator that regulates glucose and lipid homeostasis; physical activity targets PPARα, decreasing it’s expression, further resulting in decreased levels of VEGF, a key regulator of metastasis (Wang et al., 2006; Peters et al., 2011).

## Circadian Rhythm Alteration and Increased Risk of CRC

A convincing body of evidence suggests that there are severe repercussions of circadian disruption resulting in physiological and patho-physiological consequences. This is not surprising, considering that in last 2.5 billion years during which life has evolved on the planet, there has been a predictable rhythm between the rotating surfaces of the earth and the sun. The cells of the most organisms reveal circadian rhythms known as the circadian clock; 24-h timekeeping in the central clock of the brain and the peripheral tissues. This circadian clock modulates transcription-translation feedback loops that are generated by core circadian clock genes, including *PER1, 2*, *and 3*, and *CLOCK* (Ko and Takahashi, 2006). Suppression of these genes occurs during CRC promotion and progression; coupled with the fact that *Per2* knock out mice are associated with a tumor prone-phenotype, indicates the important role this 24-h clock plays during carcinogenesis (Fu et al., 2002; Sjoblom et al., 2006). Correspondingly, each organism has evolved mechanisms to adjust to the solar day and deal with cycles of energy overload during the day and cycles of dearth at night; protecting essential machinery from excess solar, chemical, and biochemical energy. In addition, biological timing of DNA damage repair, cellular proliferation, and apoptosis are under the control of the circadian rhythm (Hrushesky et al., 1998; Granda et al., 2005; Kang et al., 2009). Furthermore, considering women and men that are chronically exposed to night-shift work, have a 50% increased risk of developing CRC compared to day shift workers; understanding this circadian time structure and the biological targets provides unique opportunities to prevent colon carcinogenesis (Hrushesky et al., 2009). Identifying molecular targets whose expression is controlled by the circadian clock is key when designing prevention strategies for CRC.

**Figure 5:**
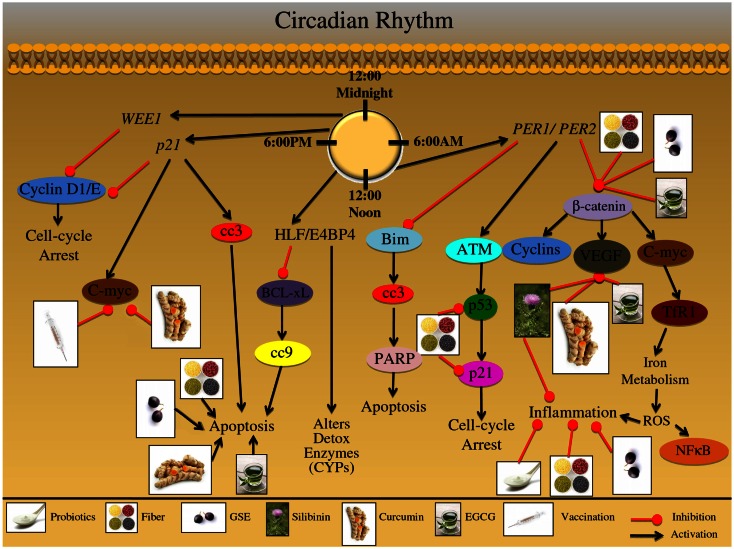
**Effect of circadian rhythm on growth and development of colon carcinogenesis**. The mammalian circadian clock takes 24 h to complete and is a self-sustaining feedback loop of core clock genes. This group of clock genes regulate various cellular processes including: detoxification; DNA repair; proliferation; cell cycle regulation; apoptosis; metastatic signaling, and inflammation. Furthermore, these cellular processes are then regulated in a time specific manner; disruption of this circadian rhythm results in abnormalities in these processes, which contribute to colorectal cancer (CRC) development. The major genes regulating this 24 h timekeeping are *PER1* and *PER2*; these genes modulate proliferative, apoptotic, inflammatory, and metastatic signaling. These genes have been shown to alter the expression of β-catenin; this protein is involved in proliferation and cell cycle regulation. Additionally, β-catenin can modulate oncogenic proteins such as C-myc, which have been shown to regulate iron metabolism; iron metabolism results in ROS formation, further triggering an inflammatory response and NFκB activation. Moreover, β-catenin can modify growth and metastatic signals, mediated via VEGF protein upregulation. Another protein that is under the control of *PER1*/*PER2* genes is ATM, which is up regulated in response to cellular stress; this stress regulator protein can further modify downstream targets, such as p53. The p53 protein, known as the guardian of the genome, can up regulate p21 protein levels resulting in cell cycle arrest; the arrest allows the cell to repair the DNA damage that has occurred due to cellular stress. The *PER1*/*PER2* gene complex further inhibits apoptotic signaling; specifically through Bim protein inhibition, which results in down regulation of cleaved-caspase-3 (cc3) and PARP. Alternatively, a group of binding proteins which are regulated in a circadian manner, including the proteins HLF and E4BP4 can further modulate detoxification of enzymes, such as cytochrome P450 subenzyme (CYPs). In addition HLF/E4BP4 binding proteins inhibit the apoptotic process through inhibition of the pro-apoptotic protein BCL-xL; BCL-xL inhibition results in down regulation of cleaved-caspase-9 (cc9). Additional genetic regulators of the circadian process are *p21* and *WEE1*; these genes are involved in numerous cellular processes including proliferation, cell cycle regulation, and oncogene signaling. Both *WEE1* and *p21* regulate Cyclin D and Cyclin E expression; these cyclin proteins are involved in cell cycle regulation, which is abnormally regulated during CRC development. Additionally, *p21* genetic regulation can lead to the induction of cellular apoptotic signals via cc3 induction; *p21* can further regulate oncogenic proteins such as C-myc, which is an important protein in the development and progression of CRC. The illustration also denotes the modulatory role of lifestyle interventions at various levels on these aberrant signaling pathways. The photographs of lifestyle interventions were sourced and adapted from http://www.freedigitalphotos.net/.

A review of the circadian rhythm pathways that are involved in CRC etiology is shown in Table 1 and Figure 5. The circadian clock also controls pathways involved in detoxification enzyme expression, such as CYPs; furthermore, it has been shown that these detoxification genes are expressed in a rhythmic pattern throughout the GI tract (Hoogerwerf et al., 2007; Sladek et al., 2007). Proline and acidic amino acid-rich (PAR) basic leucine zipper (bZip) transcription factors are also circadian clock-controlled, and form a complex with hepatic leukemia factor (HLF) or E4 promoter binding protein-4 (E4BP4); this group of transcription factors are responsible for xenobiotic detoxification (Murakami et al., 2008). Moreover, the rhythmic regulation of *p21/Wee1* genes controls numerous physiological processes, which gate other growth and cell cycle regulators including DNA replication and mitosis (Bjarnason et al., 2001; Matsuo et al., 2003). Additionally, elimination of the *Per2* gene in mice resulted in lack of p53 protein activity, which is involved in DNA repair, cell cycle, and cell death pathways (Fu et al., 2002). Cancer cells also express higher levels of *PER2* compared to normal cells; correspondingly, *PER2* circadian expression has been shown to modulate proliferative, cell cycle, and apoptotic pathways, specifically c-myc, cyclin D, β-catenin, and VEGF (Zhang et al., 2001; Fu et al., 2002; Wood et al., 2008). Iron is an important mineral involved in metabolism, respiration and DNA synthesis; iron is regulated through transferrin receptor 1 (TfR1) which binds and internalizes transferrin and also exhibits a 24-h rhythm (Sorokin et al., 1989). Furthermore, iron metabolism and immunity are closely related; TNF-α and IL-6 inflammatory cytokines directly stimulate iron storage proteins (Brock and Mainou-Fowler, 1986; Torti et al., 1988; Rogers, 1996).

## Biological Targets of Nutraceuticals for CRC Prevention

**Figure 6:**
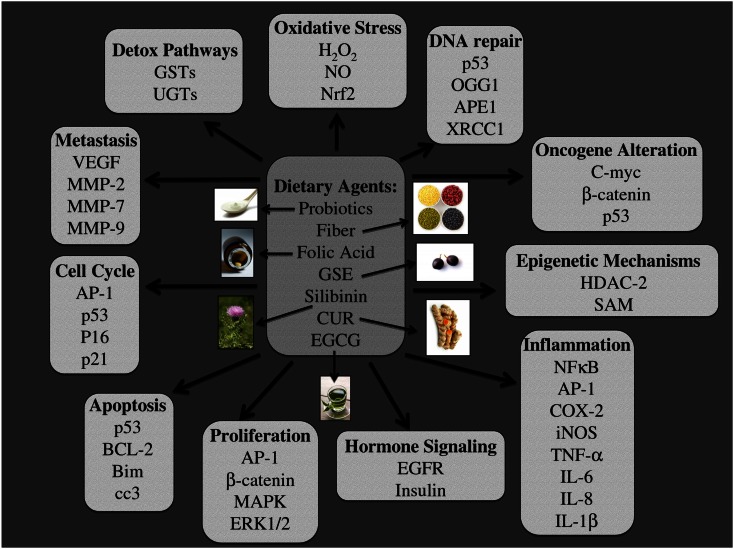
**Major molecular targets of dietary agents involved in their efficacy against colon carcinogenesis**. Multiple signaling pathways, which are modified due to dietary intervention using probiotics, fiber, folic acid, grape seed extract (GSE), silibinin (Sb), curcumin (CUR), and Epigallocatechin-3-gallate (EGCG) are listed. The specific pathways involved are those related to, detoxification, oxidative stress, DNA repair, oncogenesis, epigenetic silencing, inflammation, hormone signaling, proliferation, apoptosis, cell cycle, and metastasis. The photographs of dietary agents were sourced and adapted from http://www.freedigitalphotos.net/.

A healthy diet is important in maintaining basic physiological functions within the body. With that in mind, the ACS has recommended an intake of ≥5 serving of fruits and vegetables and further limiting the consumption of refined grains, sugars, fats, and red meat. Fruits and vegetables are part of a large plant kingdom that is a vast source of phytochemicals; a number of these compounds have been investigated for their anti-cancer and chemoprevention efficacy (Ramos, 2008). Furthermore, probiotic therapy has been shown to exert health benefits and these beneficial organisms further increase the production of various vitamins such as folic acid. Lifestyle interventions based on natural sources not only have the potential to prevent carcinogenesis, but also are beneficial in maintaining good health because they are natural sources of vitamins, minerals, and fiber. Furthermore, these nutraceuticals make ideal chemopreventive agents due to their multi-targeted effects and selective toxicity toward cancer cells. These nutraceuticals are also efficacious against various forms of cancer, can be administered by oral route, are accepted by the general public, and are produced at a low cost (Galati and O’Brien, 2004). Furthermore, nutraceutical administration can target numerous cellular processes, all of which are important regulators during the development of CRC as outlined in Figure 6.

### Probiotic consumption

Residing in the GI tract are a variety of microorganisms comprising of bacteria, archaea, viruses, and unicellular eukaryotes, which are know to augment a variety of immunological and metabolic pathways (Licciardi et al., 2010; Vipperla and O’Keefe, 2012). Collectively, there are over 800 species and 7000 strains that form over 100-trillion super colonies, which are influenced by several factors including diet, environment, stress, and disease (Burk et al., 2003; Vipperla and O’Keefe, 2012). Intestinal microbial composition has become an emerging factor in CRC susceptibility, since these organisms impact multiple physiological functions that are related to cancer progression, cell proliferation, differentiation, metabolism of essential nutrients, and stimulation of intestinal immunity; similarly, probiotics have been shown to prevent carcinogenesis through a variety of mechanisms (Zhu et al., 2011; Kumar et al., 2013). The fields of nutrition, microbiology, and genomics are converging rapidly, providing insight into the molecular mechanisms involved in the chemopreventive efficacy of probiotics; further fostering the development of inexpensive preventive therapies for CRC. The development of probiotic-based CRC prevention therapy has great therapeutic implications, due to its ability to target multiple pathways. Probiotic multi-targeted approach results in increased DNA repair and detoxification enzymes; furthermore, probiotic consumption inhibits multiple processes including DNA mutation, DNA-adduct formation, growth signaling, insulin signaling, and inflammation as shown in Figures 1–6.

*Saccharomyces boulardii* is a non-pathogenic yeast species that has demonstrated antioxidant, metabolic, anti-toxin, and anti-inflammatory effects (Czerucka et al., 1994; Castagliuolo et al., 1996; Dalmasso et al., 2006). Compounds produced by the intestinal microbiota, specifically polyamines, have been shown to reduce oxidative stress (Noack et al., 1998; Rhee et al., 2007). Several mechanisms by which probiotics may suppress CRC development have been suggested, including induction of the adaptive immune response through increased production of inflammatory cytokines, such as TNF-α and IL-6; further modulating downstream detoxification pathways (CYPs, GSTs, COX-2) and reducing the uptake of carcinogenic compounds (Rafter, 2004). Mechanistic studies indicate that *S. boulardii* modulates the host-pathogen interaction; specifically through ERK1/2 MAPK pathway, which is down regulated in both *in vitro* and *in vivo* studies (Dahan et al., 2003; Chen et al., 2006b). Modulation of the MAPK pathway can lead to altered hormone, inflammatory, apoptotic, proliferative, and metastatic signals. *Bifidobacterium animalis* also increases polyamine concentrations and has been shown to alter IGF-1 expression (Matsumoto et al., 2011). Similarly, *Bacillus polyfermenticus*, also known to possess chemopreventive efficacy, has been shown to modulate inflammatory and proliferative signals, such as those associated with EGFR pathway (Kim et al., 2006; Lee et al., 2007; Park et al., 2007). The ErbB receptor family, including EGFR, is important in cancer development and these family members are over expressed in various cancers including CRC (Holbro et al., 2003; Kamath and Buolamwini, 2006). ErbBs also regulate the cell cycle effecting cyclin D1 levels and further downstream oncogenic targets, such as c-myc (Slansky and Farnham, 1996; Rafter, 2004).

### Dietary fiber consumption

Epidemiological studies indicate that diets rich in fruits, vegetables, and legumes are of great importance when considering CRC prevention strategies; these foodstuffs are rich in soluble dietary fiber (DF) and insoluble fibers, starches, oligosaccharides, and phenolic compounds (Michels, 2005; Vergara-Castaneda et al., 2010). These DFs are fermented via microorganisms in the colon resulting in several physiological effects: production of short chained fatty acids (SCFA), butyrate, propionate, and acetate; furthermore, these compounds have been shown to induce apoptosis in human CRC cells (Chen et al., 2006a; Campos-Vega et al., 2009). Potential mechanisms associated with fiber consumption and reduced CRC risk include: dilution of carcinogens, reduction of transit time, production of SCFAs, and reduction of tumor-promoting substances. Further identifying these molecular mechanisms, induced after fiber consumption, plays an important role in CRC prevention.

Dietary fiber was shown to decrease levels of deoxycholic acid (DCA), which is a bile acid produced via the dehydroxylation of primary bile acids and is known to be a direct as well as indirect tumor promoter in the colon (Zampa et al., 2004; Stein et al., 2012). Similarly, butyrate induces GSTs in human CRC cells, which results in carcinogen detoxification (Pool-Zobel et al., 2005). Furthermore, DF acts as an anti-inflammatory agent decreasing the levels of IL-6 and TNF-α; additional studies have also shown the ability of DF to inhibit *COX-2* and *iNOS* gene expression (Reddy et al., 2000; Kaczmarczyk et al., 2012). Additionally, DF intake was associated with the increased expression of DNA repair genes, specifically *Apex*, *Xrcc4*, and *Xrcc5*, which bind to DNA ligase initiating the repair of DSB (Vergara-Castaneda et al., 2010). Similarly, butyrate induces apoptotic signaling in CRC cells through up regulation of BAK, cleaved caspase-3 and cleaved PARP, and loss of mitochondrial function (Ruemmele et al., 1999). Furthermore, other SCFA found in legumes altered the expression of β*-catenin*, *p53*, *p21*, *Bax*, and *casp3* genes in a carcinogen induced CRC model; all of which are important modulators of cell cycle, proliferation, and cell death (Feregrino-Perez et al., 2008). Butyrate also alters epigenetic pathways through HDAC inhibition in CRC cells (Waldecker et al., 2008). This alteration leads to hyperacetylation of histone residues altering gene transcription and expression; for example HDAC inhibition via SCFAs results in upregulation of *p21*, which is involved in cell cycle, proliferation, differentiation, and apoptosis (Chen et al., 2004).

### Folic acid supplementation

Folates are water-soluble B vitamins that are important cofactors in DNA synthesis and methylation pathways. Humans need to consume folic acid from exogenous sources to support these essential functions; good sources of folates include leafy greens, vegetables, yeast extracts, and citrus fruits (Lucock, 2000). Studies indicate a 40–60% reduction in the risk of CRC in individuals with high folate intake (Kim, 1999, 2007). One of the potential mechanisms in CRC development and progression is folate deficiency (FD); low levels lead to DNA strand breaks, impaired DNA methylation, and repair (Kennedy et al., 2011). CRC patients frequently have decreased folate levels; this deficiency is recognized as one of the metabolic stressors of colorectal carcinogenesis (Martinez et al., 2004). The mechanisms and protein targets by which folate conveys protection against CRC development need to be investigated to develop strategies that effectively target and prevent colon carcinogenesis. Furthermore, supplementation with folic acid has clinical potential; this is due to its ability to target multiple pathways that are involved in CRC initiation, promotion, and progression. Folic acid supplementation results in increased DNA repair proteins, as well as, inhibits multiple processes including DNA mutation, epigenetic silencing, DNA-adduct formation, growth signaling, metastatic signaling, and inflammatory processes as shown in Figures 2–4 and 6.

Folate deficiency may play a role in chronic inflammation through activation of NF-κB and TNF-α, perpetually creating inflammatory signals (Wang et al., 2012b). Furthermore, it has been reported that DNA repair genes are up regulated in folate-depleted cells, specifically *Xrcc5*, *Apex*, and PCNA, supporting folic acids’ role in DNA repair and synthesis (Duthie et al., 2008; Sadik and Shaker, 2012). Additionally, PCNA plays a role in proliferative pathways, as well as, in DNA repair, indicating an additional mechanism of action in which folic acid is protective against CRC development. Folate supplementation studies reveal its ability to increase the stability of p53, which is involved in DNA repair, proliferation, and cell death pathways (Sadik and Shaker, 2012). Studies have also elucidated FD role in epigenetic modifications; its deficiency was associated with decreased global DNA methylation. Furthermore, different folate species are used for methylation versus DNA synthesis; these species are distributed differentially in normal colon tissue compare to cancer tissues (Liu et al., 2012a). In addition, FD has been shown to enhance EMT signaling in HCT116 colon cancer cells, through activation of MMPs, up regulation of *Snail*, and suppression of *E-cadherin* (Wang et al., 2012b).

### Nutritional supplementation with grape seed extract

Another supplement that has shown promising chemopreventive and anti-cancer potential in numerous types of cancer, including skin, prostate, breast, lung, and CRC, is grape seed extract (GSE) (Kaur et al., 2009a). GSE supplementation has vast chemopreventive potential; this is due to its ability to target multiple proteins that are involved in the development of CRC. GSE supplementation results in increased cancer cell apoptosis, as well as, it inhibits multiple processes such as signaling related to epigenetics, growth, proliferation, oncogenes, insulin, metastasis, and inflammation as shown in Figures 1–6.

Grape seed extract is a complex mixture of polyphenols, specifically proanthocyanidins. *In vivo* studies with GSE have shown its anti-inflammatory effects reducing the expression of iNOS and COX-2 (Velmurugan et al., 2010b). Additionally, GSE has also been shown to decrease global methylation, and it also decreases DNA-methytransferase (DNMT) activity and protein levels, indicating GSE’ role during epigenetic gene modification (Vaid et al., 2012). Furthermore, GSE inhibited aberrant β-catenin expression and downstream proteins, cyclin D1, and c-myc (Velmurugan et al., 2010b). Moreover, mechanistic studies of GSE, at various stages of CRC development, identified apoptosis induction as the major factor in the chemopreventive efficacy of GSE against CRC; specifically it induces caspase-3, -8, -9 resulting in the cleavage of PARP, DNA fragmentation, and PCD (Derry et al., 2012). The apoptotic effect was specific to CRC cells, with minimal effect on normal colon epithelial cells; furthermore, this effect was attenuated with antioxidant treatment, indicating ROS as a potential upstream stimulus in GSE-induced CRC cell death (Derry et al., 2012). In addition, GSE has been shown to modulate p21 levels; resulting in decreased proliferation, leading to cell cycle arrest, and further downstream pathway activation, including that of ERK1/2 (Kaur et al., 2006, 2011).

### Herbal supplementation with silibinin

Silibinin is a flavonolignan constituent of silymarin, which is extracted from the milk thistle plant (*Silybum marianum*). It is non-toxic and has been used traditionally as well as clinically to treat various liver diseases and liver complications (Post-White et al., 2007; Ramasamy and Agarwal, 2008). In past two-decades, however, silibinin has been investigated for its cancer chemopreventive properties; this natural non-toxic agent is found efficacious in *in vivo* preclinical studies against skin, lung, prostate, bladder, and CRC cancer models (Rajamanickam and Agarwal, 2008; Ramasamy and Agarwal, 2008). Several *in vitro* and *in vivo* studies have shown strong preventive and/or therapeutic efficacy of silibinin against CRC growth and progression (Agarwal et al., 2003; Rajamanickam and Agarwal, 2008; Velmurugan et al., 2008, 2010a; Kaur et al., 2009b, 2010; Rajamanickam et al., 2009, 2010; Ravichandran et al., 2010; Raina et al., 2013a,b). Silibinin herbal supplementation already has potential against CRC; this is due to its ability to target multiple proteins that are involved in the initiation, promotion, and progression of CRC. Silibinin supplementation results in increased levels of detoxification enzymes; it also inhibits multiple processes in carcinogenesis, such as those involved in DNA mutation, growth/proliferation, metastatic signaling, and inflammation, as shown in Figures 1–6.

Mechanistic investigations have revealed that the anti-CRC effects of silibinin were mainly due to its anti-proliferative and pro-apoptotic effects; further, silibinin strongly inhibits the Wnt/β-catenin pathway, which is known to play an essential role in the development and progression of CRC (Rajamanickam and Agarwal, 2008; Kaur et al., 2009b, 2010; Rajamanickam et al., 2009). Toward this effect, silibinin strongly inhibited β-catenin dependent transcriptional activity of Tcf-4 in CRC cells, which was followed with a decrease in the expression of its transcriptional targets namely, cyclin D1 and c-myc (Kaur et al., 2010). Furthermore, the pro-apoptotic effects of silibinin are via the down regulation of the anti-apoptotic proteins BCL-2, MCL-1, XIAP, and survivin and up regulation of pro-apoptotic proteins such as Bax (Velmurugan et al., 2010a; Kauntz et al., 2012a). Additional studies have shown that silibinin up regulates death receptors, DR4/DR5, at the transcriptionally level; these receptors are involved in the extrinsic apoptotic pathway, which further supports the ability of silibinin to induced apoptotic death as an additional mechanism involved in its chemopreventive efficacy (Kauntz et al., 2012b). Studies have also revealed that silibinin strongly inhibits PI-3K-AKT–mTOR but activates MEK1/2-ERK1/2 pathways for its biological effects manifested in terms of induction of autophagic type PCD in CRC cells (Raina et al., 2013a). Silibinin is also known to cause endoplasmic reticulum stress in CRC cells together with glucose uptake inhibition as well as energy restriction, due to interference in essential cellular processes such as mitochondrial metabolism, phospholipid and protein synthesis; the cellular damage to CRC cells by silibinin is severe and irreparable (Raina et al., 2013a). Importantly, it is non-toxic to normal colon cells (Rajamanickam et al., 2009, 2010). Raina et al. (2013b) have also shown that silibinin has the potential to strongly inhibit TNFα-induced NF-κB activation in human CRC cells. These observations were also corroborated by *in vivo* studies which showed that anti-inflammatory mechanisms of silibinin are associated with decreased expression of COX-2 and iNOS levels, together with inhibition of NF-κB transcriptional activity (Rajamanickam et al., 2009, 2010; Ravichandran et al., 2010; Raina et al., 2013b) Moreover, other studies have shown that silibinin reduced inflammatory mediators such as, TNF-α and IL-1β; further modifying downstream target proteins, such as MMP-7 (Kauntz et al., 2012a). Likewise, silibinin alters numerous pathways that are involved in cell invasion and metastasis via alteration of the JNK pathway and down regulation of AP-1, resulting in decreased MMP-2 levels (Lin et al., 2012). Silibinin has been shown to alter xenobiotic metabolizing enzymes; specifically, reducing CYP2E1 activity which is involved in metabolic activation of carcinogens, and in contrast, silibinin upregulated Phase II enzymes including, GSTs, which are important in metabolic detoxification (Sangeetha et al., 2012). Importantly, Hoh et al. (2006) in a completed pilot study with silibinin in CRC patients with colon adenocarcinoma have shown high bioavailability of silibinin in colonic tissue of CRC patients. Thus, the ongoing research on the usefulness of silibinin has strong translational implications in the prevention and management of CRC.

### Curcumin dietary supplementation

Curcumin (CUR) is the active phenolic compound extracted from the spice turmeric, derived from the rhizome *Curcuma longa*. CUR has been shown to possess anti-cancer and chemopreventive efficacy, and in clinical trials, CUR is effective in reducing CRC burden (Hatcher et al., 2008). CUR dietary supplementation has the potential to target multiple signaling pathways that are involved in the development of CRC. CUR consumption results in increased cancer cell apoptosis, and levels of detoxification enzymes; it also inhibits multiple processes including that of DNA mutation and signaling related to growth, metastasis, and inflammation, as shown in Figures 1–6.

Numerous studies have examined pharmacological effects of CUR in CRC such as induction of apoptosis, and inhibition of proliferation, oxidative stress and angiogenesis (Shehzad et al., 2010). CUR has been shown to up regulate GST enzymes in multiple studies; furthermore, CUR has been shown to induce ROS in cancer cells, resulting in p21 up regulation and G2/M cell cycle arrest (Ye et al., 2007; Odenthal et al., 2012; Yogosawa et al., 2012). CUR has also been shown to alter MAPK pathway resulting in decreased expression of TNF-α and COX-2 expression in colon mucosa, resulting in an overall decrease in inflammation (Camacho-Barquero et al., 2007). Additionally, CUR has been shown to down regulate NF-κB and IL-6 secretion, which are also key regulators of the chronic inflammatory response (Tu et al., 2012). Concentrated analogs of CUR have been also shown to inhibit NF-κB translocation, thus resulting in a down regulation of gene targets, such as *c-myc*, *cyclin D1*, and *BCL-2* (Chen et al., 2011). CUR also induces mitochondrial stress and apoptotic cell death through up regulation of Bax, Bim, Bak, Puma, and Noxa, and down regulation of anti-apoptotic proteins such as BCL-2 and BCL-xl (Basile et al., 2013). This induced mitochondrial dysfunction may be due to the ability of CUR to induce ER-stress resulting in accumulation of ubiquitinated proteins; this protein accumulation could also induce autophagy (Basile et al., 2013). Furthermore, CUR analogs were able to decrease VEGF and MMP-9 expression, indicating the ability of CUR to prevent metastatic disease (Chen et al., 2011).

### Dietary intervention with EGCG

Tea is a one of the worlds most consumed beverages made from the leaves of *Camellia sinensis*; Epigallocatechin-3-gallate (EGCG) is the major polyphenol. Tea has been cultivated and used medicinally for thousands of years due to the beneficial health effects. Furthermore, it has been extensively studied for its chemopreventive efficacy (Bode and Dong, 2009; Yang et al., 2009). Dietary intervention with EGCG has great anti-CRC potential; this is due to its ability to target multiple proteins that are involved in the development of CRC, as shown in Figures 1–6.

A number of potential mechanisms have been proposed for the chemopreventive efficacy of EGCG including: enhancement of antioxidant activity, alteration of hormone and growth factor signaling, and induction of cell cycle arrest. Due to the polyphenolic structure of EGCG, tea polyphenols are strong antioxidants and thus prevent the formation of ROS. Similarly, EGCG has been shown to initiate transcription factors in CRC cells, such as Nrf2, which further induces phase II enzymes such as UDP-glucuronosyltransferases (UGTs) (Zhang et al., 2009). Furthermore, EGCG decreases growth and hormone signals specifically EGF, IGF-1, and VEGF indicating its role in proliferation, insulin, and metastatic signaling (Khan and Mukhtar, 2008). In addition, EGCG inhibits additional proliferative and cellular division pathways in CRC cells, via down regulation of MAPK/ERK1/2 and p21 pathways, ultimately resulting in cell cycle arrest (Lin et al., 1999; Larsen and Dashwood, 2010). Moreover, EGCG has the ability to induce apoptotic death of cancer cells via p53, p21, and PUMA up regulation (Thakur et al., 2010). EGCG can also modify epigenetic expression patterns in CRC cells via alteration of DNMT activity, resulting in gene silencing (Fang et al., 2003). EGCG was also observed to inhibit β-catenin translocation and VEGF expression, which further supports its chemopreventive efficacy (Kondo et al., 2002).

## The Role of Vaccination for CRC Prevention

Chronic inflammatory conditions have been shown to increase the incidence of CRC (Terzic et al., 2010). The question still remains: does inflammation precede cancer or does cancer precede inflammation. During chronic inflammation, the tissue environment contains a diverse population of leukocytes that secrete and express factors that effect cell proliferation and genomic instability (Balkwill et al., 2005; Lin and Karin, 2007). Antigen presenting cells such as dendritic cells (DCs) have the ability to acquire antigens and migrate to lymph nodes, which can lead to T-cell stimulation, specifically CD4^+^ T helper cells and CD8^+^ cytotoxic T lymphocytes (CTLs). These cells can be hijacked for anti-tumor activity; furthermore, vaccines have been designed to present antigens against cancer cells, causing immune cell infiltration in the tumor microenvironment, and resulting in decreased tumor growth. This immune recognition is mediated by toll-like receptors (TLRs) which bridge the innate and adaptive immunity (Iwasaki and Medzhitov, 2004). Specific cancer antigens including carcinoembryonic antigen (CEA), are utilized clinically to monitor CRC disease progression and is one of the most promising tumor associated antigens (TAAs) (Thompson et al., 1991). The theory behind cancer vaccination is that priming of the innate immunity to neoantigens may clear the tissue site and prevent chronic inflammation, or with tumor specific neoantigens, vaccination could prevent cancer progression. There have been several clinical trials investigating vaccination for CRC, DC based vaccines in particular, that have shown to induce a specific immune response (Mosolits et al., 2005). Furthermore, vaccination has great clinical potential with treatment resistant patient sub-types; this is due to its ability to target specific proteins that are involved in the progression of CRC. Vaccination inhibits multiple processes that are involved in the development of metastatic CRC including growth and oncogenic signaling, and inflammatory processes, as shown in Figures 1,2,4, and 5.

Tumor vaccines have been designed to target oncogenes, specifically c-myc, which is over expressed in 80% of CRC; vaccination resulted in generation of CD4^+^ and CD8^+^ T cells that infiltrated the tumor site (Williams et al., 2008). Additionally, these have been designed to target other mediators, such as TGF-β which plays a role in inflammatory, proliferative, and apoptotic pathways; vaccination resulted in inhibition of tumor growth (Roberts, 2002). Furthermore, CRC vaccines have been designed to target epithelial specific proteins such as MUC1, which is over expressed in chronic inflammatory diseases such as, inflammatory bowel disease; resulting in an increased innate immune response and decreased chronic inflammation (Beatty et al., 2007; Furr et al., 2010). Consequently, the anti-tumor efficacy of the MUC1 vaccine was mediated by CD4^+^ T cells, FasL, and TNF-α signaling, resulting in induction of the innate immunity and apoptosis (Sugiura et al., 2008). Clinically, anti-VEGF (Bevacizumab) vaccines are currently utilized, however, adverse effects have been observed, and therefore, different vaccination formulations are being designed against VEGF with anti-metastatic potential and no toxicity, supporting a role for vaccination in the prevention of CRC (Rad et al., 2007).

## Lifestyle Recommendations

Consumption of red meat is standard in the normal American diet; however, due to the numerous associations between red meat consumption and CRC risk, it is recommended to limit the consumption of red meat to 50 g/day, which is equivalent to 2 oz, about 1/2 the size of your palm. Additionally, consuming other foodstuffs along with red meat could be beneficial in decreasing the detrimental effects of red meat consumption. For example, probiotic consumption alters heme metabolism and reduces inflammation. Probiotics, fiber, silibinin, and CUR have the ability to increase DNA repair enzymes; again, neutralizing the detrimental effects of high red meat consumption. Furthermore, nutraceuticals such as GSE, silibinin, and CUR have all been shown to possess anti-inflammatory effects, indicating that these agents would be beneficial in inhibiting red meat-induced inflammation.

Alcohol is considered a carcinogen, and therefore, to prevent CRC, daily intake of alcohol should be kept to 20 g/day or below to be considered as safe exposure, which is equivalent to approximately two glasses of wine or beer (Haas et al., 2012). Additionally, other measures can be taken to limit toxicity. Probiotics not only can increase DNA repair enzymes, but these organisms also produce folic acid, which is a chronic nutrition deficient in individuals that consume alcohol on a regular basis. In addition, folic acid supplementation has been shown to decrease the incidence of DNA mutations that may result following alcohol exposure. Furthermore, alcohol consumption has been shown to increase proliferation; in contrast fiber, GSE, silibinin, CUR, and EGCG have been shown to alter β-catenin signaling resulting in decreased proliferation rates. Downstream targets of β-catenin signaling include MMP-7, which is involved in CRC metastatic disease progression. Nutraceuticals such as silibinin and CUR have been shown to decrease MMPs expression, specifically MMP-2 and MMP-7, indicating the ability of these supplements to decrease the detrimental effects that result from chronic alcohol exposure.

Tobacco smoke contains nicotine and numerous carcinogenic compounds that result in colon carcinogenesis, and there are no safe exposure guidelines for tobacco smoke. Therefore, for preventing CRC, individuals should not smoke tobacco, and additional lifestyle modifications should be considered. Specifically, foodstuffs that target carcinogen detoxification through up regulation of GSTs and DNA repair proteins would be extremely beneficial; these include probiotics, folic acid, fiber, and phytochemicals such as, silibinin and CUR. Additionally, tobacco smoke exposure results in epigenetic modifications of *KRAS* and *BRAF*, and consumption of fiber, GSE, or EGCG has the ability to inhibit these epigenetic changes. Similarly, the epigenetic modifications resulting from tobacco exposure can initiate alterations in proliferative and apoptotic signaling cascades, resulting in uncontrolled proliferation and apoptosis resistance. To counteract this, it would be beneficial to consume EGCG, which has been shown to inhibit aberrant β-catenin signaling, inhibit proliferation, and inflammatory signaling. Furthermore, nutraceuticals such as, GSE, silibinin, fiber, EGCG, and CUR have all been shown to induce apoptotic cancer cell death. In addition, probiotics, GSE, silibinin, CUR, and EGCG all produce anti-inflammatory effects, which would be beneficial in inhibiting the inflammatory mediators that are up regulated in response to tobacco smoke and contribute to CRC disease promotion and progression. Coupled with the fact that silibinin has also been shown to inhibit VEGF levels, these dietary factors have enormous potential for CRC prevention as a result of tobacco exposure.

Obesity has become a major epidemic in the US, and obesity-related conditions such as cancer are on the rise as well. The most significant lifestyle intervention that would prevent CRC development and be further beneficial to overweight and obese individuals would be increased physical activity. This change in lifestyle would result in decreased adipose tissue and thus decrease angiogenic and inflammatory mediators that are released from the fat tissue. In addition, physical activity has the ability to prevent chronic inflammation, through inhibition of TNFα and induction of the innate immune response. Moreover, physical activity also increases the release of hormones that regulate satiety signals, ultimately restoring the balance between energy intake and expenditure. However, there are a number of diet alterations that can also modify obesity-related signals, specifically, GSE, silibinin, probiotics, and EGCG have all been shown to beneficially modify insulin signaling. Like physical activity, additional nutraceuticals can alter GST activation; these include probiotics, fiber, silibinin, and CUR, all of which result in increased detoxification. Obesity-induced CRC is also related to uncontrolled proliferation rates, which also could be modified through fiber, GSE, and EGCG supplementation. Furthermore, consumption of silibinin or CUR could decrease VEGF levels, which are elevated in obese individuals, further inhibiting CRC disease progression and metastasis.

Maintaining a regular daily schedule that coincides with the circadian rhythm is important in preventing cancer development including CRC. Furthermore, as a proof of principle, these clock genes are suppressed during CRC promotion and progression. It would be recommended to work day shifts; however, that may not be an option for everyone. If night-shift work is necessary, supplementing with compounds that decrease inflammation and control the cell cycle could alleviate the damaging effects of circadian rhythm disruption. Specifically, probiotics, fiber, GSE, silibinin, CUR, EGCG, and specific vaccinations all decrease inflammation. Moreover, fiber, GSE, silibinin, CUR, and EGCG modulate p21 protein, which causes cancer cell cycle arrest as well as inhibition of proliferation.

Overall, the above lifestyle choices can result in damaging or beneficial effects, the key is to find a balance. Furthermore, increasing the protective lifestyle factors is associated with decreased rates and incidence of CRC. The current knowledge highlights the multiple pathways and molecular targets involved in lifestyle choices that can result in CRC progression or prevention. This comprehensive review sheds light on the etiology and pathogenesis of CRC, and provides additional evidence that lifestyle modifications are important of the prevention of CRC.

## Conflict of Interest Statement

The authors declare that the research was conducted in the absence of any commercial or financial relationships that could be construed as a potential conflict of interest.
